# Antibiotic exposure and growth patterns in preterm, very low birth weight infants

**DOI:** 10.1186/s40748-021-00126-6

**Published:** 2021-01-29

**Authors:** Alaina K. Pyle, Joseph B. Cantey, L. Steven Brown, Roy J. Heyne, Phillip S. Wozniak, Elizabeth Heyne, Amy Holcombe, Elizabeth M. Brammer, Cheryl S. Lair, Pablo J. Sánchez

**Affiliations:** 1 Department of Pediatrics, University of Texas Southwestern Medical Center, Dallas, TX USA; 2 Department of Pediatrics, Division of Neonatology, Connecticut Children’s Medical Center, Hartford, CT USA; 3 Department of Pediatrics, Divisions of Neonatology and Pediatric Infectious Diseases, University of Texas Health Science Center San Antonio, San Antonio, TX USA; 4 Health System Research, Parkland Health and Hospital System, Dallas, TX USA; 5 Department of Pediatrics, Divisions of Neonatology and Pediatric Infectious Diseases, Center for Perinatal Research, Abigail Wexner Research Institute at Nationwide Children’s Hospital, Nationwide Children’s Hospital, The Ohio State University College of Medicine, Columbus, OH USA; 6 Children’s Medical Center, Dallas, TX USA; 7 Nutrition Services, Parkland Health and Hospital System, Dallas, TX USA

**Keywords:** Very low birth weight, Neonate, Antibiotic, Obesity, Stewardship

## Abstract

**Background:**

Antibiotic exposure in term infants has been associated with later obesity. Premature, very-low-birth-weight (birth weight ≤ 1500 g) infants in the neonatal intensive care unit frequently are exposed to antibiotics. Our hypothesis was that in preterm infants, there is a positive linear and dose-dependent relationship between antibiotic exposure and growth from birth through 12 months’ corrected age.

**Methods:**

Retrospective analysis of prospectively collected data of all antibiotic use among inborn, preterm (≤32 weeks’ gestation), very-low-birth-weight infants admitted to the neonatal intensive care unit at Parkland Memorial Hospital and followed in the Low Birth Weight Clinic at Children’s Medical Center, Dallas, TX. Antibiotic use was quantified by days of therapy which was compared with weight and length parameters at birth, 36 weeks’ postmenstrual age, and 2, 4, 6, and 12 months’ corrected age. The change in weight and length z-scores from birth to all subsequent age points was calculated. Stepwise multivariate regression analysis was performed to determine predictors of weight, length, and weight-for-length delta z-scores from birth to each subsequent age point.

**Results:**

During the 18-month study, 161 infants received a median of 11 (IQR, 5.5–19.5) antibiotic days of therapy which was not associated with weight or length delta z-scores from birth through 12 months’ corrected age.

**Conclusion:**

Association of prolonged antibiotic use and neonatal morbidities and mortality may override the potential association with increased weight gain in the NICU and beyond.

## Background

Antibiotic exposure in early infancy has been associated with later obesity, presumably from alteration of the intestinal microbiome that leads to decreased bacterial biodiversity and modulation of host metabolism [1, 2]. Antibiotic therapy also may result in long-lasting perturbations of metabolic pathways, thereby increasing the risk for metabolic syndrome and increased adiposity in adult life [3–5]. Although such outcomes have been associated with low birth weight [6–8], the impact of antibiotic exposure in the neonatal intensive care unit (NICU) on growth among preterm infants is not known. Therefore, the objective of this study was to determine the effect of antibiotic use on weight and growth patterns in preterm, very-low-birth-weight (VLBW, birth weight ≤ 1500 g) infants in the NICU. We hypothesized that during a period of critical bacterial microbiome development, there is a positive linear and dose-dependent relationship between antibiotic exposure and growth from birth through 12 months’ corrected age.

## Methods

This study was a retrospective analysis of prospectively collected data of all antibiotic use among inborn, preterm (≤32 weeks’ gestation), and VLBW infants admitted to the NICU at Parkland Memorial Hospital, Dallas, TX, from 7/1/2011 to 12/31/2012 and subsequently followed in the Low Birth Weight Follow-up Clinic at Children’s Medical Center (CMC), Dallas as their medical home. Antibiotic use was quantified by days of therapy (DOT) which was determined by multiplying the number of antibiotic doses by the dosing interval, then dividing by 24 h (i.e., 4 doses of ampicillin administered every 8 h = 1.33 DOT) [9]. DOT were compared with weight and length parameters that were obtained at birth, 36 weeks’ postmenstrual age, and approximately 2, 4, 6, and 12 months’ corrected age.

Pertinent clinical, laboratory, microbiologic, and outcome data were collected on all infants from the electronic health record. Severity of illness was calculated for all infants using the Clinical Risk Index for Babies (CRIB)-II score, a validated risk-adjustment instrument for prediction of mortality among VLBW infants that incorporates gestational age, birth weight, sex, temperature on admission, and initial base deficit [10]. CRIB-II scores range from 0 to 27 with higher scores predictive of greater mortality risk. For example, scores of 0, 5, 10, and 15 predict a mortality risk of 0.2, 1.4, 12.2, and 56.8%, respectively. The daily weight of each infant was obtained by the bedside nurse and weekly length measurement was performed by the NICU dieticians using a standardized measuring board. In addition, the following data were obtained on each infant during the NICU stay: total number of days that the infant was NPO which was defined as ≤1 feeding per 24-h period, total number of days that the infant received total parenteral nutrition, and the caloric density of enteral feeds that the infant received at 36 weeks’ postmenstrual age. A standardized feeding protocol was utilized throughout the study period, and there was no use of donor human milk.

Intrauterine growth restriction (IUGR) was defined as a ponderal index < 10% for gestational age [11] while small for gestational age (SGA) was defined as birth weight < 10th percentile. Sepsis was defined as a positive culture of a clinically relevant bacterial or fungal pathogen from a normally sterile site and for which the infant received an antimicrobial agent for ≥5 days [12]. Sepsis was considered to be early or late-onset if it occurred at ≤ or > 72 h postnatal age, respectively. Bronchopulmonary dysplasia (BPD) was defined as need for oxygen at 36 weeks’ postmenstrual age [13]. The inclusion of patent ductus arteriosus (PDA) in the analysis required confirmation by echocardiography and treatment with indomethacin or surgical closure. Necrotizing enterocolitis (NEC) was defined as modified Bell stage IIa or greater [14].

Weight and length z-scores at birth and 36 weeks’ postmenstrual age were calculated using the 2013 Fenton growth chart [15]. The WHO Anthropometric calculator was used to calculate weight-for-length z-scores, adjusted for gestational age, from weight and length measurements obtained on follow-up visits at 1–3 months’, 3–5 months’, 5–8 months’, and 10–14 months’ chronologic age, which corresponded to pediatric care provider visits at approximately 2, 4, 6, and 12 months’ corrected age [16]. The change in weight z-score and length-z-score from birth to 36 weeks’ postmenstrual age, and birth to 2, 4, 6, and 12 months’ corrected age was then calculated.

### Statistical analyses

Descriptive analyses, including mean and standard deviation or median and interquartile range, were performed as indicated. All tests were two-tailed, and a *p*-value < 0.05 was considered significant. Univariate linear regression was used to evaluate the relationship between change in z-score for weight and length from birth to each subsequent age point and demographic and clinical variables, including days of antibiotic therapy. A stepwise multivariate regression model was performed for weight, length and weight-for-length delta z-scores at each age point. Pearson correlations were calculated for all variables; *p*-value < 0.05 was considered significant. To control for severity of illness, bronchopulmonary dysplasia, patent ductus arteriosus, necrotizing enterocolitis, and late-onset sepsis were included in the multivariate analysis. Since the study was a secondary analysis of a prospective evaluation of all antibiotic use in the NICU, a post hoc power analysis was performed that showed that a sample size of 126 patients produced a two-sided confidence interval with a lower limit of 0.13 and an upper limit of 0.45 when the sample correlation was 0.30.

The study was approved by the Institutional Review Board of the University of Texas Southwestern Medical Center with waiver of informed consent.

## Results

**Fig. 1:**
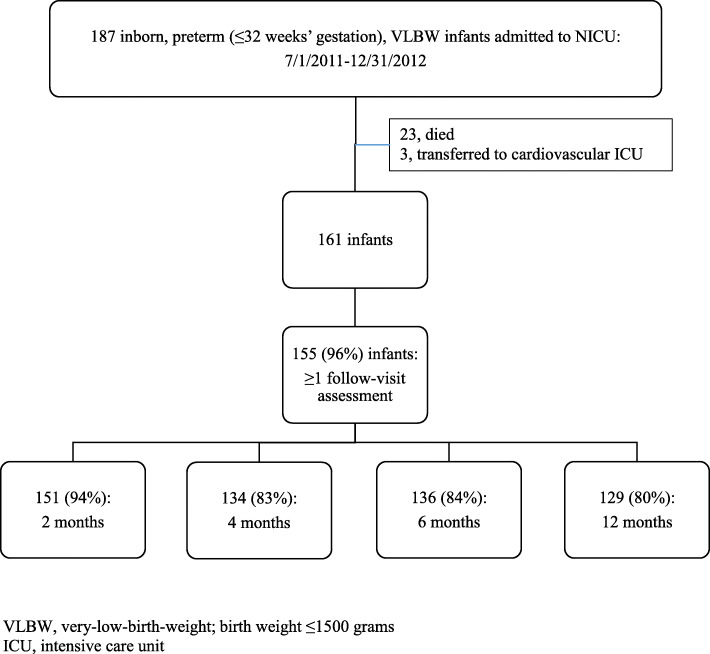
Study population

During the 18-month study period, 187 inborn, preterm, VLBW infants were admitted to the NICU (Fig. 1). Of these 187 infants, 23 died and 3 were transferred to the cardiovascular intensive care unit at CMC before 36 weeks’ postmenstrual age and were excluded from the study. The clinical characteristics of the remaining 161 infants are shown in Table 1. The majority of infants were singleton, born by cesarean delivery, appropriate for gestational age, and did not have PDA, NEC, BPD, or late-onset sepsis. None of the infants had early-onset sepsis. Of the 161 infants, 157 received at least 48 h of antibiotics and the median antibiotic DOT received was 11 (IQR, 5.5–19.5).

**Table 1:** Characteristics of the study population

Number of infants	161
**Gestational age (weeks, mean ± SD)**	28.5 ± 1.97
**Delivery by caesarean section**	124 (77%)
**Singleton, twin, triplet gestation**	117 (73%), 39 (24%), 5 (3%)
**Male sex**	81 (50%)
**Birth weight (grams; mean ± SD)**	1130 ± 229
**Birth length (cm; mean ± SD)**	37 ± 3
**Ponderal index (mean ± SD)**	2.22 ± 0.28
**Intrauterine growth restriction**	14 (9%)
**Small for gestational age**	9 (6%)
**Clinical Risk Index for Babies-II score (mean ± SD)**	7.4 ± 2.9
**Antibiotic exposure (median; IQR)**^**a**^	11 (5.5–19.5)
**Bronchopulmonary dysplasia**	29 (18%)
**Patent ductus arteriosus**	40 (25%)
**Necrotizing enterocolitis (Bell stage ≥ 2)**	8 (5%)
**Late-onset sepsis**	1 episode, 19 (12%); 2 episodes, 9 (6%)

*SD* standard deviation, *cm* centimeter, *IQR* interquartile range

^a^Antibiotic exposure defined as days of therapy (DOT)

Of the 161 infants, 155 (96%) had at least 1 follow-up visit to the CMC Low Birth Weight Follow-up clinic. Complete weight and length data were available for 151 (94%) infants at ~ 2-month visit, 134 (83%) infants at ~ 4 months, 136 (84%) infants at ~ 6 months, and 129 (80%) infants at ~ 12 months. Weight alone was available for an additional 1 infant at 2 months, 1 infant at 4 months, 3 infants at 6 months, and 1 infant at 12 months.

The weight and length z-scores at birth were within the standard range for gestational age by the Fenton curves, with the average weight and length z-scores being 0.074 ± 0.91 and 0.096 ± 0.93, respectively. The weight and length z-scores decreased by an entire standard deviation from birth to 36 weeks’ corrected age, to − 0.98 ± 0.92 and − 1.18 ± 0.82, respectively. After 36 weeks’ corrected age, there was a steady increase toward a normal average z-score of zero for both weight and length, although the length z-score remained lower than the weight z-score. At 2 months’ corrected age, there was an elevated weight-for-length z-score of 0.997 ± 1.08 but by 12 months’ corrected age, the average weight-for-length z-score approached zero.

Univariate linear regression was performed to evaluate the impact of antibiotic exposure as well as demographic features and clinical diagnoses on growth patterns (Tables 2, 3). There was a negative correlation between antibiotic exposure and weight delta z-score at only 36 weeks’ and 2 months’ corrected age. A negative correlation with weight z-scores also was seen at 36 weeks’ corrected age in males, at 2 months’ corrected age for late-onset sepsis, at 36 weeks, 2 months, and 4 months for total days of TPN, and at 36 weeks and 2 months for total days NPO. CRIB-II score and BPD had a negative relationship with change in weight z-score at multiple time points (Table 2). A positive correlation with weight z-scores was seen in infants who were SGA, especially at 4, 6, and 12 months’ corrected ages. Gestational age showed a persistently strong positive correlation with weight delta z-score, indicating that infants born later in gestation had improved catch-up weight gain through the first-year post-term.

**Table 2:** Weight delta z-scores from birth to subsequent age points by univariate linear regression analysis^a^

	Weight Delta Z-score at Age of Evaluation				
	~ 36 weeks	~ 2 months	~ 4 months	~ 6 months	~ 12 months
**No. of infants**	161	151	134	136	129
**Gestational age**	0.274^b^	0.477^b^	0.446^b^	0.445^b^	0.479^b^
**Mode of delivery**	0.270^b,c^	0.177^b,c^	0.121	0.130	0.111
**Multiple gestation**	0.091	0.081	0.034	0.022	0.025
**Sex**	−0.208^d,e^	− 0.111	− 0.072	− 0.091	− 0.040
**IUGR**	0.052	0.194^b^	0.163	0.164	0.162
**SGA**	0.059	0.078	0.191^b^	0.247^b^	0.250^b^
**CRIB-II score**	−0.206^d^	−0.379^d^	− 0.334^d^	− 0.310^d^	−0.321^d^
**Antibiotic exposure (days of therapy [DOT])**	−0.375^b^	−0.295^b^	− 0.151	−0.080	− 0.119
**BPD**	−0.294^d^	− 0.262^d^	−0.195^d^	− 0.127	−0.209^d^
**PDA**	−0.138	−0.107	− 0.077	−0.053	− 0.112
**NEC**	−0.174	− 0.023	0.051	0.069	0.035
**Late-onset sepsis**	−0.074	−0.192^d^	− 0.129	−0.052	− 0.075
**TPN (total days)**	−0.397^d^	− 0.356^d^	−0.206^d^	− 0.131	−0.127
**Total days of NPO**	−0.367^d^	−1.313^d^	−0.177	− 0.092	−0.109
**Caloric density (36 weeks, kcal/ounce)**	−0.024	0.066	0.022	0.018	0.006

*IUGR* intrauterine growth restriction, *SGA* small for gestational age, *CRIB* Clinical Risk Index for Babies, *BPD* bronchopulmonary dysplasia, *PDA* patent ductus arteriosus, *NEC* necrotizing enterocolitis, *TPN* total parenteral nutrition, *NPO* nothing per oral

^a^Pearson correlation

^b^Positive correlation, *P* < 0.05

^c^Positive correlation with delivery by caesarean section

^d^Negative correlation, *P* < 0.05

^e^Negative correlation with male sex

**Table 3:** Length delta z-score from birth to subsequent age points by univariate linear regression analysis^a^

	Length Delta Z-score at Age of Evaluation				
	~ 36 weeks	~ 2 months	~ 4 months	~ 6 months	~ 12 months
**No. of infants**	161	151	134	136	129
**Gestational age**	0.042	0.281^b^	0.329^b^	0.222^b^	0.251^b^
**Delivery mode**	0.159^b,c^	0.191^b,c^	0.118	0.052	0.073
**Multiple gestation**	0.015	0.076	0.061	0.042	0.086
**Sex**	−0.069	−0.116	− 0.014	− 0.018	− 0.016
**IUGR**	0.253^d^	0.091	0.041	0.070	0.043
**SGA**	0.106	0.039	0.219^b^	0.283^b^	0.291^b^
**CRIB-II score**	0.263	0.244^d^	0.232^d^	0.130	0.118
**Antibiotic exposure (days of therapy [DOT])**	0.021	−0.144	−0.092	−0.040	−0.077
**BPD**	−0.120	−0.216^d^	− 0.217^d^	−0.138	− 0.130
**PDA**	−0.153	− 0.171^d^	−0.151	0.008	0.018
**NEC**	−0.020	0.015	−0.002	0.073	0.003
**Late-onset sepsis**	0.244^b^	−0.019	−0.056	− 0.049	0.007
**TPN (total days)**	0.033	−0.216^d^	−0.159	− 0.073	−0.112
**Caloric density (36 weeks, kcal/ounce)**	−0.070	0.003	0.012	0.015	0.081

*IUGR* intrauterine growth restriction, *SGA* small for gestational age, *CRIB* Clinical Risk Index for Babies, *BPD* bronchopulmonary dysplasia, *PDA* patent ductus arteriosus, *NEC* necrotizing enterocolitis, *TPN* total parenteral nutrition

^a^Pearson correlation

^b^Positive correlation, *P <* 0.05

^c^Positive correlation with delivery by caesarean section

^d^Negative correlation, *P <* 0.05

Factors associated with a change in length z-score from birth to each subsequent age-point also were assessed. Antibiotic exposure had no relationship with length delta z-score at any age point. IUGR, CRIB-II score, BPD, PDA and total days of TPN all showed small negative relationships for at least one of the early age points: 36 weeks’, 2 months’ and/or 4 months’ corrected age. Gestational age and SGA had a positive relationship with length delta z-score at all age points except 36 weeks’ corrected age, indicating that infants born at older gestational ages or SGA had improved catch-up growth over the first year of age.

Stepwise multivariate regression analysis was performed to predict weight delta z-scores from birth to each subsequent age point using the variables that were significant on at least one time point by univariate analysis (antibiotic exposure, gestational age, sex, delivery mode, IUGR, SGA, CRIB-II score, BPD, late-onset sepsis, total days of TPN, and total days NPO; Table 4). Antibiotic exposure was not associated with weight delta z-scores at any age point in the multivariate model. The strongest positive predictor for weight at all age points was length with the converse also true. Gestational age remained a moderate predictor of weight but not length. Total duration of TPN was a strong negative predictor for weight delta z-score from birth to 36 weeks’ corrected age. The change in z-score for weight, length, and weight-for-length between each consecutive age point had no significant predictors (data not shown).

**Table 4:** Weight ^a^ and length ^b^ delta z-scores from birth to subsequent age points by stepwise multivariate regression analysis^c^

	Delta Weight z-Score^a^				
	~ 36 weeks	~ 2 months	~ 4 months	~ 6 months	~ 12 months
**No. of infants**	161	151	134	136	129
**Length**	0.359	0.476	0.562	0.599	0.606
**Gestational age**	–	0.346	0.242	0.272	0.316
**IUGR**	0.137	0.174	0.138	0.151	0.119
**Total parenteral nutrition (total days)**	−0.349	–	–	–	–
**Delivery mode**	0.174	–	–	–	–
**Late-onset sepsis**	–	−0.153	–	–	–
**PDA**	–	0.143	–	–	–
	**Delta Length z-Score**^**b**^				
	**~ 36 weeks**	**~ 2 months**	**~ 4 months**	**~ 6 months**	**~ 12 months**
**Weight delta z-score**	0.444	0.568	0.644	0.674	0.701
**Late-onset sepsis**	0.152	–	–	–	–

^a^The total days of parenteral nutrition showed a strong negative correlation with weight z-scores at 36 weeks’ postmenstrual age, while positive associations were seen with gestational age, length, and IUGR status. Antibiotic exposure was not significant at any time point. *P-*value was ≤0.05 for all displayed z-score values.

^b^Positive associations were seen with the length delta z-score and late-onset sepsis (*p <* 0.05).

^c^Pearson correlation

*IUGR* intrauterine growth restriction, *PDA* patent ductus arteriosus

## Discussion

Unlike other studies that have associated antibiotic use in term infants, older children, and animals with increased weight, length, and body mass index, [1, 2, 17–19] there was no correlation between antibiotic exposure and changes in weight or length z-scores from birth through 12 months’ corrected age among preterm VLBW infants in the NICU in this study. The strongest predictor of increased weight gain at all age points was higher gestational age, a finding that remained significant after controlling for severity of illness and clinical factors. This study also demonstrated the overall poor weight and length gain for VLBW infants when compared to in-utero growth patterns, but the only variable associated with a significant negative change in weight z-scores was duration of TPN use.

The substantial decrease in weight and length percentiles during the NICU hospitalization is a well-documented phenomenon in premature infants, and it is believed to be due to inadequate nutritional intake in combination with co-morbidities such as BPD, NEC, late-onset sepsis, and PDA which increase metabolic demand [20, 21]. Inadequate nutrition leading to poor growth in VLBW infants has been associated with neurodevelopmental impairment [20, 22]. Both SGA and appropriate for gestational age VLBW infants who are below the 10th percentile for height or weight at 2 years of age have significantly lower mental development (MDI) and psychomotor development indices (PDI) scores as well as higher rates of cerebral palsy [23]. Ehrenkranz et al. [20] demonstrated that in-hospital growth velocity was associated significantly with growth after discharge, cerebral palsy, MDI < 70, and PDI < 70 at 18–22 months’ corrected age. Despite antibiotics being among the most frequently prescribed medications in the NICU, [24–26] they did not augment either the weight or length of preterm infants in this study. Similarly, the lack of improved or rapid weight gain in our patients makes the contribution of antibiotics to development of metabolic syndrome unlikely, although longer term follow- up is required to fully evaluate this outcome [27].

The lack of correlation between antibiotic exposure and weight gain may be due to low overall use of antibiotic therapy as measured by DOT or the type of antibiotics used [1, 28, 29].We previously have shown that antibiotic DOT was significantly less in the study inborn NICU at Parkland Memorial Hospital than in our Level 4 outborn NICU at CMC, although comparison with other similar inborn NICUs has not been performed [9, 30, 31]. Nonetheless, the majority of infants (98%) received at least 48 h of antibiotic therapy in the NICU and while it is possible that even short duration of antibiotic exposure could have affected growth parameters, comparison to infants who never received antibiotic therapy was not possible. The more common use of ampicillin and gentamicin than the third-generation cephalosporin and carbapenem agents may have affected the results of this study. It would be important to determine whether similar findings are seen in NICUs with higher antibiotic use and exposure to broader spectrum antimicrobial agents.

One of the limitations of this study is its retrospective analysis which precludes any determination of a causal relationship between antibiotic exposure and alteration in growth parameters. Nevertheless, the information on antibiotic use was obtained prospectively and included every agent used during the study period. Moreover, the optimal metric to study antibiotic use in the NICU is not known, and it is possible that other measures may yield different results. However, DOT has been used in adult and neonatal populations and encompasses not only the days that the infant received antibiotic therapy, but the quantity utilized. The study also did not account for antibiotic therapy that the infant may have received after discharge from the NICU. Another limitation was that complete weight and length data were available for only 84 and 80% of infants at ~ 6 and 12 months’ corrected age, respectively, and longer follow-up may be necessary to detect alterations in growth percentiles especially with respect to obesity. In addition, there currently is no universally accepted and validated measure for body mass index, weight-for-length, or ponderal index in the neonatal population [32]. Strengths of the study include use of a standardized protocol for initiation and advancement of enteral feeds as well as TPN use throughout the study period that should minimize the effect of clinician variability on overall growth patterns. However, information on such variables as timing of initial enteral feeding, use of maternal milk or formula in the NICU and after discharge home, and composition of TPN was not addressed. Finally, since this was a single center study in the United States, results may not be generalizable to other NICUs or populations especially given practice changes in antibiotic stewardship that have evolved from when the study was performed.

## Conclusion

In conclusion, among the study population of VLBW infants ≤32 weeks’ gestational age, the study hypothesis of a linear and dose-dependent relationship between antibiotic exposure and weight or length z-scores from birth through 12 months’ corrected age was not confirmed. The previously described negative association of prolonged antibiotic use and increased risk of NEC, late-onset sepsis, BPD, retinopathy of prematurity, invasive fungal disease, neurodevelopmental impairment, and death may override any potential benefit with improved weight gain in the NICU and beyond [33–39].

## Data Availability

The datasets used and/or analyzed during the current study are available from the corresponding author on reasonable request.

## Abbreviations

NICU: Neonatal intensive care unit
VLBW: Very-low-birth-weight
NEC: Necrotizing enterocolitis
PDA: Patent ductus arteriosus
BPD: Bronchopulmonary dysplasia
TPN: Total parenteral nutrition
NPO: Nothing per os
PMA: Post menstrual age
DOT: Days of therapy
CRIB: Clinical Risk Index for Babies
CMC: Children’s Medical Center
IUGR: Intrauterine growth restriction
SGA: Small for gestational age
MDI: Mental developmental index
PDI: Psychomotor developmental index

## References

[1] Bailey LC, Forrest CB, Zhang P, Richards TM, Livshits A, DeRusso PA (2014). Association of antibiotics in infancy with early childhood obesity. JAMA Pediatr.

[2] Ajslev TA, Andersen CS, Gamborg M, Sorensen TI, Jess T (2011). Childhood overweight after establishment of the gut microbiota: the role of delivery mode, pre-pregnancy weight and early administration of antibiotics. Int J Obes.

[3] Cox LM, Yamanishi S, Sohn J, Alekseyenko AV, Leung JM, Cho I (2014). Altering the intestinal microbiota during a critical developmental window has lasting metabolic consequences. Cell..

[4] Cho I, Yamanishi S, Cox L, Methe BA, Zavadil J, Li K (2012). Antibiotics in early life alter the murine colonic microbiome and adiposity. Nature..

[5] Barker DJ, Eriksson JG, Forsen T, Osmond C (2002). Fetal origins of adult disease: strength of effects and biological basis. Int J Epidemiol.

[6] Frankel S, Elwood P, Sweetnam P, Yarnell J, Smith GD (1996). Birthweight, body-mass index in middle age, and incident coronary heart disease. Lancet..

[7] Hales CN, Barker DJ, Clark PM, Cox LJ, Fall C, Osmond C (1991). Fetal and infant growth and impaired glucose tolerance at age 64. BMJ..

[8] Parkinson JR, Hyde MJ, Gale C, Santhakumaran S, Modi N (2013). Preterm birth and the metabolic syndrome in adult life: a systematic review and meta-analysis. Pediatrics..

[9] Cantey JB, Wozniak PS, Sanchez PJ (2015). Prospective surveillance of antibiotic use in the neonatal intensive care unit: results from the SCOUT study. Pediatr Infect Dis J.

[10] Parry G, Tucker J, Tarnow-Mordi W, Group UKNSSC (2003). CRIB II: an update of the clinical risk index for babies score. Lancet..

[11] Lubchenco LO, Hansman C, Boyd E (1966). Intrauterine growth in length and head circumference as estimated from live births at gestational ages from 26 to 42 weeks. Pediatrics..

[12] Stoll BJ, Hansen N, Fanaroff AA, Wright LL, Carlo WA, Ehrenkranz RA (2002). Late-onset sepsis in very low birth weight neonates: the experience of the NICHD neonatal research network. Pediatrics..

[13] Ehrenkranz RA, Walsh MC, Vohr BR, Jobe AH, Wright LL, Fanaroff AA (2005). Validation of the National Institutes of Health consensus definition of bronchopulmonary dysplasia. Pediatrics..

[14] Kliegman RM, Walsh MC (1987). Neonatal necrotizing enterocolitis: pathogenesis, classification, and spectrum of illness. Curr Probl Pediatr.

[15] Fenton TR, Kim JH (2013). A systematic review and meta-analysis to revise the Fenton growth chart for preterm infants. BMC Pediatr.

[16] de Onis M, Habicht JP (1996). Anthropometric reference data for international use: recommendations from a World Health Organization expert committee. Am J Clin Nutr.

[17] Murphy R, Thompson JM, Mitchell EA (2013). Group ABCs. Early antibiotic exposure and body mass index in children born small for gestational age. Acta Paediatr.

[18] Trasande L, Blustein J, Liu M, Corwin E, Cox LM, Blaser MJ (2013). Infant antibiotic exposures and early-life body mass. Int J Obes.

[19] Gough EK, Moodie EE, Prendergast AJ, Johnson SM, Humphrey JH, Stoltzfus RJ (2014). The impact of antibiotics on growth in children in low and middle income countries: systematic review and meta-analysis of randomised controlled trials. BMJ..

[20] Ehrenkranz RA, Dusick AM, Vohr BR, Wright LL, Wrage LA, Poole WK (2006). Growth in the neonatal intensive care unit influences neurodevelopmental and growth outcomes of extremely low birth weight infants. Pediatrics..

[21] Brown LD, Hay WW (2013). The nutritional dilemma for preterm infants: how to promote neurocognitive development and linear growth, but reduce the risk of obesity. J Pediatr.

[22] Frank L, Sosenko IR (1988). Undernutrition as a major contributing factor in the pathogenesis of bronchopulmonary dysplasia. Am Rev Respir Dis.

[23] Latal-Hajnal B, von Siebenthal K, Kovari H, Bucher HU, Largo RH (2003). Postnatal growth in VLBW infants: significant association with neurodevelopmental outcome. J Pediatr.

[24] Clark RH, Bloom BT, Spitzer AR, Gerstmann DR (2006). Reported medication use in the neonatal intensive care unit: data from a large national data set. Pediatrics..

[25] Tripathi N, Cotten CM, Smith PB (2012). Antibiotic use and misuse in the neonatal intensive care unit. Clin Perinatol.

[26] Hsieh EM, Hornik CP, Clark RH, Laughon MM, Benjamin DK, Smith PB (2014). Medication use in the neonatal intensive care unit. Am J Perinatol.

[27] Kerkhof GF, Willemsen RH, Leunissen RW, Breukhoven PE, Hokken-Koelega AC (2012). Health profile of young adults born preterm: negative effects of rapid weight gain in early life. J Clin Endocrinol Metab.

[28] Saari A, Virta LJ, Sankilampi U, Dunkel L, Saxen H (2015). Antibiotic exposure in infancy and risk of being overweight in the first 24 months of life. Pediatrics..

[29] Rajpal DK, Klein JL, Mayhew D, Boucheron J, Spivak AT, Kumar V (2015). Selective Spectrum antibiotic modulation of the gut microbiome in obesity and diabetes rodent models. PLoS One.

[30] Shipp KD, Chiang T, Karasick S, Quick K, Nguyen ST, Cantey JB (2016). Antibiotic stewardship challenges in a referral neonatal intensive care unit. Am J Perinatol.

[31] Cantey JB, Wozniak PS, Pruszynski JE, Sanchez PJ (2016). Reducing unnecessary antibiotic use in the neonatal intensive care unit (SCOUT): a prospective interrupted time-series study. Lancet Infect Dis.

[32] Olsen IE, Lawson ML, Ferguson AN, Cantrell R, Grabich SC, Zemel BS, et al. BMI Curves for Preterm Infants. Pediatrics. 2015;135(3):e572-81. 10.1542/peds.2014-2777 25687149

[33] Kuppala VS, Meinzen-Derr J, Morrow AL, Schibler KR (2011). Prolonged initial empirical antibiotic treatment is associated with adverse outcomes in premature infants. J Pediatr.

[34] Cotten CM, Taylor S, Stoll B, Goldberg RN, Hansen NI, Sanchez PJ (2009). Prolonged duration of initial empirical antibiotic treatment is associated with increased rates of necrotizing enterocolitis and death for extremely low birth weight infants. Pediatrics..

[35] Cantey JB, Pyle AK, Wozniak PS, Hynan LS, Sanchez PJ (2018). Early antibiotic exposure and adverse outcomes in preterm, very low birth weight infants. J Pediatr.

[36] Novitsky A, Tuttle D, Locke RG, Saiman L, Mackley A, Paul DA (2015). Prolonged early antibiotic use and bronchopulmonary dysplasia in very low birth weight infants. Am J Perinatol.

[37] Ting JY, Roberts A, Sherlock R, Ojah C, Cieslak Z, Dunn M, et al. Duration of Initial Empirical Antibiotic Therapy and Outcomes in Very Low Birth Weight Infants. Pediatrics. 2019;143(3):e20182286. 10.1542/peds.2018-2286 30819968

[38] Ting JY, Synnes A, Roberts A, Deshpandey A, Dow K, Yoon EW (2016). Association between antibiotic use and neonatal mortality and morbidities in very low-birth-weight infants without culture-proven Sepsis or necrotizing Enterocolitis. JAMA Pediatr.

[39] Ting JY, Synnes A, Roberts A, Deshpandey AC, Dow K, Yang J (2018). Association of Antibiotic Utilization and Neurodevelopmental Outcomes among extremely low gestational age neonates without proven Sepsis or necrotizing Enterocolitis. Am J Perinatol.

